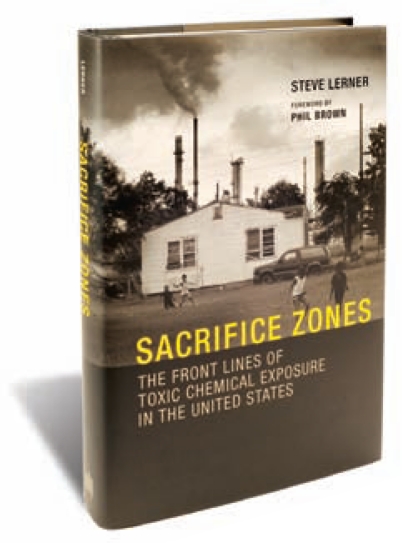# Sacrifice Zones: The Front Lines of Toxic Chemical Exposure in the United States

**Published:** 2011-06

**Authors:** Robert D. Bullard

**Affiliations:** *Robert D. Bullard directs the Environmental Justice Resource Center at Clark Atlanta University. He has written 15 books, including* The Quest for Environmental Justice: Human Rights and the Politics of Pollution *(2005),* Growing Smarter *(2007), and* Race, Place, and Environmental Justice After Hurricane Katrina *(2009).*

Steve Lerner has written a compelling treatise on why the dominant environmental protection paradigm should be overhauled to emphasize prevention, precaution, and equal protection. *Sacrifice Zones* is a significant complement to three decades of environmental justice research; it provides irrefutable empirical evidence that not all American communities are created equal. Over the course of 2 years, the author traveled to 12 communities from New York to Alaska to collect stories from residents who live in communities that are on the front line and in the middle of toxic “sacrifice zones”—some of the most polluted and poisoned places in America.

Sacrifice zones are often “fenceline communities” of low-income and people of color, or “hot spots” of chemical pollution where residents live immediately adjacent to heavily polluted industries or military bases. Quite often, this pattern of unequal protection constitutes environmental racism—a pattern first challenged in the courts in a 1979 lawsuit, *Bean v. Southwestern Waste Management*, for which I served as an expert witness. *Bean* was the first lawsuit to use civil rights law to challenge environmental racism.

*Bean* also demonstrated that environmental justice was not just a “poverty thing”; this was later confirmed by dozens of empirical studies. A 2008 study (Downey L, Hawkins B, “Race, Income, and Environmental Inequality in the United States,” Sociol Perspec 51(4):759–781) found that African Americans experience such a high pollution burden that African American households with incomes between $50,000 and $60,000 live in neighborhoods that are, on average, more polluted than the average white neighborhood of households with incomes < $10,000. Lerner assesses the diverse strategies used by affected communities to win legal settlements, educate and mobilize their residents, block permits and expansion of polluting facilities, force cleanup of contamination, extract concessions and pollution reduction from fenceline industrial plants, and target enforcement by government.

*Sacrifice Zones* graphically describes what life is like for people of color and poor people who live on the “wrong side of the tracks” and in “throw-away communities” whose residents receive unequal protection, if any protection at all; such communities contain locally unwanted land uses, or LULUs, and industries deposit pollutants just outside the factory gates. Even after more than three decades of environmental justice mobilization and activism, Lerner had hundred of communities from which to choose for his study; there is no shortage of poisoned communities.

Lerner not only captures the hard, cold, sometimes depressing statistics, but he gives us a close-up and personal account of what it is like to live, work, and sometimes die in these communities. It is not surprising that half of the cases in *Sacrifice Zones* were from the South (all of which I have visited to work with local residents) and conforms to a well-established pattern I documented in *Dumping in Dixie* (Westview Press, 1990).

This disturbing pattern was recently documented in the 2007 *Toxic Wastes and Race at Twenty* study (Bullard et al., United Church of Christ), which found that people of color make up the majority (56%) of those living in neighborhoods within 2 miles of the nation’s commercial hazardous waste facilities, and nearly double the percentage in areas beyond 2 miles (30%). People of color make up 69% in neighborhoods with clustered facilities. Siting disparities were discovered in 9 of 10 U.S. EPA regions and 40 of 44 states (90%) with hazardous waste. The findings in Lerner’s case studies mirror those uncovered in the national *Toxic Wastes and Race at Twenty* report.

One of the most important indicators of an individual’s health is one’s ZIP code. *Sacrifice Zones* joins hundreds of environmental justice books that provide compelling evidence for government to take immediate action rather than waiting and watching while mostly African Americans, Hispanics, Native Americans and Alaska Natives, and working-class whites needlessly suffer. Lerner’s case studies illustrate that African Americans and other people of color at various income levels also experience higher levels of pollution when compared with their white counterparts.

Grassroots community leaders, most of whom are led by women of color and retired persons, are taking up the leadership mantle of environmental justice and health equity. Leaders in sacrifice zones have used the environmental justice movement to leverage the power imbalance between polluting industries and fenceline residents—understanding that the struggle is not a sprint but is more akin to a marathon. The case studies detail how race and class affect health outcomes. Lerner asks, why do grandmothers have to perform the work government regulators should be doing?

Finally, Lerner offers the reader an inside view of environmental justice activism—which emerged from protests in Warren County, North Carolina, in the 1980s—that fuses environmental protection, economic and social justice, public health, and civil rights and human rights. Without a doubt, these and other grassroots struggles have transformed the environmental justice movement in the United States and the global movement for environmental and economic justice and human rights.

## Figures and Tables

**Figure:**